# Subjective behavioral measures in myopic and pre-myopic children before and after the COVID lockdown

**DOI:** 10.3389/fmed.2023.1308423

**Published:** 2023-12-14

**Authors:** Cristina Alvarez-Peregrina, Alicia Ruiz-Pomeda, Clara Martinez-Perez, Francisco Luis Prieto-Garrido, Cesar Villa-Collar, Mariano Gonzalez-Perez, Ana Gonzalez-Abad, Miguel Angel Sanchez-Tena

**Affiliations:** ^1^Department of Optometry and Vision, Faculty of Optics and Optometry, Universidad Complutense de Madrid, Madrid, Spain; ^2^ISEC LISBOA-Instituto Superior de Educação e Ciências, Lisbon, Portugal; ^3^Fundación para la Investigación e Innovación Biomédica del Hospital Universitario del Henares (FIIB HHEN), Madrid, Spain; ^4^Faculty of Biomedical and Health Science, Universidad Europea de Madrid, Madrid, Spain; ^5^Training and Development Department, Alain Afflelou Óptico, Madrid, Spain

**Keywords:** myopia, childhood, COVID-19, lifestyles, pre-myopia

## Abstract

**Background:**

There are environmental factors that may contribute to the onset of myopia. This study aims to evaluate the children’s lifestyle changes before and after the COVID-19 lockdown and how they can influence their vision.

**Methods:**

The same questionnaire was administered to children aged between 5 and 7 in Spain every year in September before (2017–2019) and after the COVID-19 lockdown (2020–2021). All the children also passed a vision exam consisting of the measurement of visual acuity (VA) and determination of objective and subjective refraction. Children were classified as myopes, pre-myopes, or hyperopes. The cut-off points to define the refractive error were established according to the value of the spherical equivalent (SE): hyperopia (SE > +0,75D), myopia (SE ≤ −0,5D), or pre-myopia (−0.5D < SE ≥ +0.75D). Data analysis is performed with the SPSS 27.0 software (SPSS Inc., Chicago, Illinois).

**Results:**

In the pre-COVID period, the pre-myopes were the ones who spent the longest time outdoors, and after the COVID lockdown, there were no differences between groups. There neither were any differences in the time spent doing near-work activities between the groups in both periods (*p* > 0.05). Regarding the spherical equivalent, in the pre-COVID period, the mean value was 0.75 ± 2.09D and after the COVID lockdown, it was 0.47 ± 1.88D (*p* < 0.001).

**Conclusion:**

Pre-myopes spent more time outdoors than myopes in the pre-COVID period, while myopes spent more time using digital devices. All these differences do not exist after the COVID lockdown, with a general increase in the time spent outdoors and a decrease in the use of digital devices. Further studies are needed to know if these lifestyle changes remain and how they influence the onset of myopia.

## 1 Introduction

Myopia is a complex condition with multiple factors that contribute to its development, including genetics, environmental, and lifestyle factors (1, 2). Some environmental factors that may contribute to the onset of myopia include spending too much time on near-work activities (such as reading or using electronic devices), lack of outdoor time, and reduced exposure to natural sunlight (3–5).

The increasing prevalence and severity of myopia have become a major public health concern. It is estimated that by 2050, half of the global population will develop myopia, with 10% of these cases being high myopia (6). The rate of myopia progression can differ greatly among individuals due to various factors, such as the age of onset, genetic factors, and parental myopia, among others. Available evidence suggests that myopia progression rates appear to be age-dependent, delaying the onset of myopia is likely to slow the progression (7). Therefore, it is important to detect myopia early and start interventions as soon as possible to help prevent more severe myopia and associated complications later in life. To provide a framework for research into myopia prevention, the International Myopia Institute (IMI) has recently defined “Pre-myopia” as “a refractive state of an eye of ≤+0.75 D and >−0.50 D in children where a combination of baseline refraction, age, and other quantifiable risk factors provide a sufficient likelihood of the future development of myopia to merit preventative interventions” (2).

Pre-myopia previously identified as a public health problem in Asia (7), is also a problem in European populations. The concept of pre-myopia refers to the early stages of myopia development before a child has fully developed myopia. During this stage, the child may exhibit certain signs or risk factors that suggest they are at a higher risk of developing myopia in the future. Risk factors for pre-myopia include having a family history of myopia, spending excessive time on close-up activities such as reading or using electronic devices, and spending less time outdoors (4). There have also been some studies suggesting that during the COVID-19 quarantine, students were at risk of not getting enough outdoor time and the COVID-19 pandemic and the shift to remote learning may have contributed to an increase in myopia progression among children (8). In response to the lockdowns, approximately 80% of the world’s student population has changed their lifestyle and behavior (9, 10).

The COVID-19 quarantine in Spain began on March 14th, 2020, when the government declared a state of emergency due to the growing outbreak of the virus. The quarantine measures included home confinement, strict restrictions on mobility, and the closure of schools (11). As a result of quarantine and lockdown measures, the lifestyles of children could be impacted. After 9 weeks at home, the children were able to go outside without time limitations. In September 2020, face-to-face educational activity was resumed, adopting a series of prevention, hygiene, and health promotion measures against COVID-19 (12).

It has been reported that a higher number of near-work activities is linked to higher odds of becoming myopic (13) and there is evidence to indicate the relationship between an increase in near-work due to confinement or a decrease in time spent outdoors and a worsening of myopia during COVID-19 lockdown (14).

Prolonged exposure to screen light from electronic devices and a lack of outdoor time spent due to mobility restrictions have contributed to an increase in the incidence of myopia in children (7, 15), but further studies are needed to confirm these findings.

This study aimed to evaluate and compare lifestyles (outdoor time, near-work activities, and the percentage of use of electronic devices) of myopic, pre-myopic, and hyperopic children aged between 5 and 7 in Spain before and after the COVID lockdown.

## 2 Materials and methods

Observational, cross-sectional, prevalence, and multi-site study, carried out in all of the autonomous community regions in Spain. At the school ages of 5 and 7 years, a visual screening was carried out on those children who participated in the campaign of the Alain Afflelou Foundation called “School Campaign in favor of Children’s visual health.” This campaign targets all schools in Spain, so all children who were starting their school stage and who were interested in participating were included in the study. This way, the goal was to gather data from the children population without prior knowledge of whether they may or may not have visual issues. The recruitment of participants was on a voluntary basis and all the children who participated in the campaign were included.

The parents of all the children declared that they understood the objectives of the study and signed the informed consent. The research adheres to the principles of the Declaration of Helsinki, which was approved on April 25th, 2019, by the ethics committee of the European University of Madrid under code CIPI/19/102.

### 2.1 Clinical procedure

#### 2.1.1 Lifestyle questionnaire

Before beginning the optometric exam, parents of children must complete a questionnaire divided into five sections, which include questions about:

- Personal data: Age, sex, nationality, and place of residence.
- Main complaint: Routine check-ups, vision loss, headaches, or other reasons.
- Medical and ocular history of the patient: Date of the last review, user of glasses or contact lenses, systemic or ocular diseases, allergies, etc.
- Medical and ocular history of family members: systemic or ocular diseases, history of myopia, etc.
- Extracurricular activities: Number of hours per day outdoors, that is, the hours they were exposed to sunlight. On the other hand, there were questions about the time they spent doing tasks in near vision and using digital devices, not including school hours.
- The objective of this questionnaire was to obtain qualitative information on the geographical origin, lifestyles, and genetics of the children. All participants had to read each of the questions and mark only one possible answer with a cross.

#### 2.1.2 Vision exam

In the visual screening, objective refraction was measured through Mohindra retinoscopy, while subjective refraction was done via the method of initial maximum plus to maximum visual acuity, to learn the maximum relaxation capacity for their maximum visual acuity.

### 2.2 Definition of variables

To determine the children’s refractive error, the spherical equivalent (SE) criterion was used. The formula applied was SE = sphere + cylinder/2. Refractive errors were defined as follows: hyperopia: SE > +0.75D; myopia: SE ≤ −0.5D; pre-myopia: (−0.5D < SE ≥ +0.75D). A subdivision of myopia levels was established according to the American Academy of Optometry’s classification (16). Thus, one participant was classified as having low myopia when the value of the spherical equivalent was between −0.50D and −3.00D; moderate myope with a spherical equivalent between −3.00D and −6.00D, and high myope when it was more negative than −6.00D.

On the other hand, according to the Clinical Myopia Profile classification (17), the values of extracurricular activities were defined as:

- Hours a day that children spent outdoors: low (less than 1.6 h), medium (more than 1.6 and less than 2.7 h), and high (more than 2.7 h a day).
- Hours a day in near vision: low (less than 2 h a day), medium (more than two and less than 3 h a day), and high (more than 3 h a day). The time with electronic devices was also determined as low (less than 25% of the time in near vision), medium (higher than 25% and lower than 50% of the time in near vision activities), or high (higher than 50% of the time working at near distances).

### 2.3 Statistical analysis

The statistical analysis was carried out using SPSS 27.0 software (SPSS Inc., Chicago, Illinois). The normal distribution of the variables was tested using the Kolmogorov–Smirnov test, with a significance level equal to 0.05. Due to a nonparametric distribution, the Kruskal Wallis nonparametric test was used to analyze the quantitative variables, and the Chi-square test for the qualitative variables. To assess statistical significance, a cut-off value of *p* ≥ 0.05 was used.

## 3 Results

A total of 9,463 children participated in the study from 2017 to 2021. They were classified as pre-COVID period if the eye examinations and questionnaires were registered in 2017 and 2019 (*n* = 6,128) and COVID-years to those registered in 2020 and 2021 (*n* = 3,280), 3 and 15 months after the COVID lockdown in Spain.

Table 1 shows the mean and median age of all the participants, as well as the gender percentages. Of the total sample, 20.3% (*n* = 1918) were myopic and 79.7% (*n* = 7,538) were non-myopic.

**Table 1:** Demographic data of the study population.

	Total	Myopic	Non-myopic	*p*-value
Age (years)				**<0.001**
Mean ± SD	6.14 ± 0.79	6.25 ± 0.77	6.12 ± 0.79	
Median [IQR]	6 [1]	6 [2]	6 [2]	
Gender				0.177
Boys	6,402 (52.7%)	1,232 (53.3%)	5,170 (52.6%)	
Girls	5,747 (47.3%)	1,078 (46.7%)	4,669 (47.4%)	

Statistically significant values indicated in bold.

### 3.1 Myopes vs. non-myopes

Specifically, both groups spent more time outdoors, less time in near-work activity, and less using digital devices after the COVID lockdown compared to the pre-COVID period (all *p* < 0.001).

Time spent outdoors increased significantly after the COVID lockdown, increasing the percentage of children who spend moderate (an increase of 15.5%) and high time outdoors (a rise of 5%; *p* < 0.001). Regarding near-work activities, the percentage of children spending moderate time doing those activities increased by 15.4%, while the proportion of those spending high time decreased by 7.0% (*p* < 0.001). Finally, the percentage of children who used digital devices between 25 and 50% of the time doing near activities increased by 8.5%, while those who spent more than 50% of near-work time with digital devices were reduced by 2.8% (*p* < 0.001).

### 3.2 Myopes vs. pre-myopes vs. hyperopes

Of the whole sample, 20.3% (n = 1918) were myopes, 42.4% (*n* = 4,013) were pre-myopes, and 37.3% (*n* = 3,532) were hyperopes. The mean age of the myopes was 6.24 ± 0.77 (Median [IQR]: 6 [1]), of the pre-myopes 6.09 ± 0.80 (Median [IQR]: 6 [2]), and the hyperopes 6.13 ± 0.79 (Median [IQR]: 6 [2]). There were significant differences in the age group of myopes compared with pre-myopes and hyperopes (both *p* < 0.001). No significant differences were found in the age groups of pre-myopes and hyperopes (*p* > 0.05). As shown in Table 2, no significant differences were found regarding gender in the three groups (*p* > 0.05).

**Table 2:** Demographic data based on myopes vs. pre-myopes vs. hyperopes.

	Myopes	Pre-myopes	Hyperopes	*p*-value
Age (years)				0.247
Mean ± SD	6.24 ± 0.77	6.09 ± 0.80	6.14 ± 0.79	
Median [IQR]	6 [1]	6 [2]	6 [2]	
Gender				0.177
Boys	899 (48.0%)	2012 (49.5%)	1703 (48.3%)	
Girls	972 (52.0%)	2055 (50.5%)	1821 (51.7%)	

In terms of outdoor time, in all three groups, the amount of time increased after the COVID lockdown compared with the pre-COVID period (all *p* > 0.001). Furthermore, for all groups, there was a decrease in the number of hours spent on near-work activities and the use of electronic devices (all *p* < 0.001).

Table 3 shows the comparison of lifestyles based on gender. Resulting in that boys spend more time outdoors compared to girls (p < 0.001). Table 4 shows the differences in time spent outdoors, doing near activities, and using digital devices depending on the refractive error. When comparing each period separately, before COVID lockdown, pre-myopes were the children that spent more time outdoors. However, after COVID lockdown, there were no differences between groups (*p* > 0.05). There neither were any differences in the time spent doing near-work activities between the groups in both periods (p > 0.05). The percentage of time spent on electronic devices before COVID lockdown was higher for myopes (*p* = 0.009), while after COVID lockdown there were no differences between the groups.

**Table 3:** Comparison of lifestyle based on gender.

	Boys	Girls
Outdoor time		
Low (0–1.6 h/day)	1,511 (31.3%)	1,596 (34.8%)
Moderate (>1.6–2.7 h/day)	2,246 (46.6%)	2,197 (47.9%)
High (>2.7 h/day)	1,066 (22.1%)	797 (17.4%)
*p*-value	**<0.001**	
Near work activities		
Low (0–2 h/day)	2,167 (44.9%)	2077 (45.3%)
Moderate (>2-3 h/day)	1708 (35.4%)	1,674 (36.5%)
High (>3 h/day)	947 (19.6%)	837 (18.2%)
*p*-value	0.2	
Percentage of time spent on electronic devices		
<25%	2,166 (44.9%)	2,109 (45.9%)
25–50%	1796 (37.2%)	1,694 (36.9%)
>50%	860 (17.8%)	787 (17.1%)
*p*-value	0.533	

Statistically significant values indicated in bold.

**Table 4:** Comparison of lifestyles in the pre-COVID and post-COVID lockdown depending on the refractive error.

	Pre-COVID period			Post-COVID lockdown		
	Myopes	Pre-myopes	Hyperopes	Myopes	Pre-myopes	Hyperopes
Outdoor time						
Low (0–1.6 h/day)	491 (39.4%)	867 (36.8%)	1,103 (43.7%)	137 (20.6%)	338 (20.7%)	170 (17.2%)
Moderate (>1.6–2.7 h/day)	557 (44.7%)	1,016 (43.1%)	988 (39.1%)	381 (57.4%)	934 (57.2%)	567 (57.4%)
High (>2.7 h/day)	198 (15.9%)	474 (20.1%)	434 (17.2%)	146 (22.0%)	360 (22.1%)	251 (25.4%)
*p*-value	**<0.001**					
Outdoor time	Girls					
Low (0–1.6 h/day)	236 (40.3%)	477 (40.5%)	552 (47.0%)	67 (21.8%)	170 (20.7%)	93 (18.7%)
Moderate (>1.6–2.7 h/day)	275 (46.9%)	495 (42.1%)	457 (38.9%)	181 (59.0%)	485 (59.0%)	284 (57.1%)
High (>2.7 h/day)	75 (12.8%)	205 (17.4%)	164 (14.1%)	59 (19.2%)	167 (20.3%)	120 (24.2%)
*p*-value	**<0.001**					
Outdoor time	Boys					
Low (0–1.6 h/day)	255 (38.6%)	390 (33.1%)	551 (40.8%)	70 (19.6%)	168 (20.7%)	77 (15.7%)
Moderate (>1.6–2.7 h/day)	282 (42.7%)	521 (44.1%)	531 (39.3%)	200 (56.0%)	449 (55.4%)	283 (57.6%)
High (>2.7 h/day)	123 (18.7%)	269 (22.8%)	270 (19.9%)	87 (24.4%)	193 (23.9%)	131 (26.7%)
*p*-value	**<0.001**					
Near work activities						
Low (0–2 h/day)	563 (45.2%)	1,124 (47.7%)	1,178 (46.7%)	280 (42.2%)	680 (41.7%)	418 (42.4%)
Moderate (>2-3 h/day)	414 (33.2%)	761 (32.3%)	777 (30.8%)	289 (43.5%)	720 (44.1%)	421 (42.7%)
High (>3 h/day)	269 (21.6%)	472 (20.0%)	570 (22.6%)	95 (14.3%)	231 (14.2%)	147 (14.9%)
*p*-value	**<0.001**					
Percentage of time spent on electronic devices						
<25%	559 (44.9%)	1,116 (47.3%)	1,232 (48.8%)	254 (38.3%)	693 (42.5%)	420 (42.5%)
25–50%	428 (34.3%)	785 (33.3%)	877 (34.7%)	299 (45.0%)	686 (42.1%)	415 (42.0%)
>50%	259 (20.8%)	456 (19.3%)	416 (16.7%)	111 (16.7%)	252 (15.5%)	153 (15.5%)
*p*-value	**<0.001**					

Statistically significant values indicated in bold.

When comparing the times “pre-COVID period” and after “COVID lockdown,” with respect to outdoor activities, a decrease in the percentage of participants who spend “low” time outdoors in the three groups of refractive errors has been found. On the other hand, an increased percentage of participants who spend a “moderate” and “high” time (*p* < 0.001). Regarding near vision activities, the percentage of participants who spend a “moderate” time after the confinement period has increased in the three groups of refractive errors. On the contrary, those who spend a “low” and “high” time have decreased (*p* < 0.001). At the same time, the percentage of participants who spend between “25–50%” of the time with electronic devices has also increased in the three groups of refractive errors. On the contrary, those who spend “<25%” and “>50%” of the time after the period of confinement have decreased (*p* < 0.001).

### 3.3 Spherical equivalent

Significant differences were found between the pre-COVID and post-COVID lockdowns (p < 0.001). Specifically, the mean value was 0.75 ± 2.09 (Median [IQR]: 0.25 [2.00]) in the pre-COVID period and 0.47 ± 1.88 (Median [IQR]: 0.00 [1.25]) after COVID. The spherical equivalent was more positive the more time was spent outdoors, both in the pre-COVID period (Low: 0.84 ± 2.13 (Median [IQR]: 0.50 [2.00]); Moderate: 0.65 ± 2.07 (Median [IQR]: 0.12 [1.75]); High: 0.78 ± 2.04 (Median [IQR]: 0.25 [1.62])) and the post-COVID lockdown (Low: 0.44 ± 1.84 (Median [IQR]: 0.00 [1.00]); Moderate: 0.43 ± 1.89 (Median [IQR]: 0.00 [1.25]); High: 0.55 ± 1.89 (Median [IQR]: 0.00 [1.37]); both <0.001). However, in the pre-COVID period, no significant differences were found between those who spent little time outdoors compared with those who spent a lot of time outdoors (*p* > 0.05).

**Figure 1:**
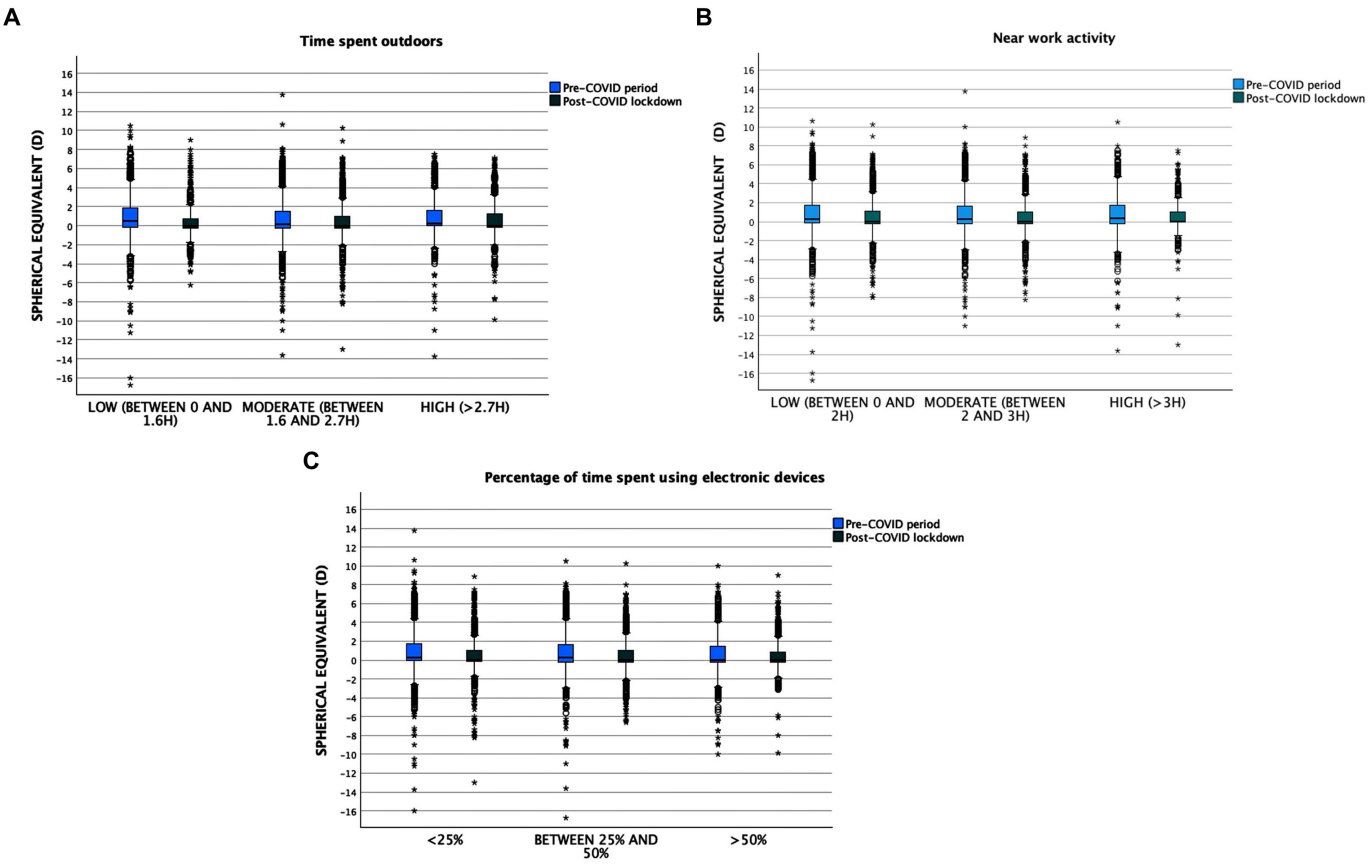
**(A)** Time spent outdoors related to spherical equivalent. **(B)** Near work activity related to spherical equivalent. **(C)** Percentage of time spent using electronical devices related to spherical equivalent.

Likewise, the higher the number of hours spent on near work activities, both in the pre-COVID period (Low: 0.78 ± 1.87 (Median [IQR]: 0.25 [1.]); Moderate: 0.71 ± 2.10 (Median [IQR]: 0.25 [1.87]); High: 0.73 ± 2.08 (Median [IQR]: 0.37 [2.00]; *p* > 0.05) and in the post-COVID lockdown (Low: 0.52 ± 1.93 (Median [IQR]: 0.00 [1.37]); Moderate: 0.41 ± 1.84 (Median [IQR]: 0.00 [1.25]); High: 0.45 ± 1.87 (Median [IQR]: 0.00 [1.06]); *p* > 0.05) and the higher the percentage of time spent on digital devices (pre-COVID (Low: 0.82 ± 2.07 (Median [IQR]: 0.25 [1.75]); Moderate: 0.73 ± 2.11 (Median [IQR]: 0.25 [1.87]); High: 0.60 ± 2.08 (Median [IQR]: 0.00 [1.75]); *p* < 0.001//COVID period (Low: 0.46 ± 1.93 (Median [IQR]: 0.00 [1.12]); Moderate: 0.46 ± 1.85 (Median [IQR]: 0.00 [1.25]); High: 0.47 ± 1.85 (Median [IQR]: 0.00 [1.12]); p > 0.05), the more negative the spherical equivalent in both periods (Figures 1A–C)

## 4 Discussion

Behavioral differences between myopic and non-myopic children have been studied previously (18), but there is little information about pre-myopic children and how habits may have influenced them during the pandemic.

There are also several studies about how COVID lockdown has affected to our lifestyles and vision, but most of them have been done just after the lockdown and with still several restrictions regarding social life and high rates of people suffering COVID.

This is the first study in children that includes visual and lifestyles data from 2 years after the Spanish lockdown (September–October 2020 and 2021) comparing the behavior before and after COVID lockdowns in pre-myopes, myopes, and hyperopes.

The study aimed to assess the refractive error and subjective measures of time spent outdoors, near-work activities, and percentage use of electronic devices after the covid-19 lockdown and to compare these results with a similar cohort examined in a pre-pandemic period. In this sense, a cross-sectional cohort study was conducted on different groups of children at the same age. Specifically, refractive data from two groups of children, one group before the pandemic and the other group after the lockdown were compared. This study design effectively controlled for the confounding effect of normal myopia progression that typically occurs with age and may provide insights into the true effect of the pandemic-related social restrictions on myopia progression. The study used questionnaires from myopic, pre-myopic, and hyperopic children to obtain subjective measures of these behaviors.

Limwattanayingyong et al. in their review highlight the significant impact of environmental and social factors on myopia development. All the cross-sectional and longitudinal questionnaire-based studies reported that there was a reduction in time spent outdoor and an increase in digital screen time during the COVID-19 lockdown (14). Our results collected 3 months and 15 months after the COVID-19 lockdown indicate that all the groups of refractive errors analyzed showed an increase in outdoor time, accompanied by a decrease in the number of hours spent on near-work activities and the use of electronic devices. We believe that the confinement and isolation marked a turning point in our way of life, making us realize the importance of being able to spend time outdoors. However, and due to the controversy between ours (19) and other studies’ results (8, 20–25) made after the lockdown and the ones collected in the present study, made a year after the lockdown, data from 2022 will be key to make strong conclusions about if lifestyles have really change in Spanish children.

In the group of myopes, time spent outdoors increased significantly after the COVID lockdown, increasing the percentage of children who spend moderate (an increase of 12.7%) and high time outdoors (a rise of 6.1%; *p* < 0.001). However, it is important to note that if we examine the different ranges, the percentage of individuals spending little time outdoors decreased (a reduction of 18.8%), but this decrease is countered by the increase in outdoor time within the “moderate” and “high” subgroups.

Our results show that in the pre-COVID era, pre-myopes were the ones who spent the most time outdoors, not finding differences among groups after the COVID. According to the Sydney Myopia Study (26), spending more than 2 h per day was associated with reduced odds of myopia even among children who spent a lot of time doing near work. Our results show that after the COVID lockdown, more than 50% of the children spent between 1.6 and 2.7 h per day outdoors. According to the latest meta-analysis, spending more time outdoors can slow down the change of axial length and decrease the risk of myopia (27); for each additional hour spent outdoors per week, the risk of developing myopia decreased by 2% (28) and the risk ratio for high versus low outdoor time was 0.54 to 0.57 in clinical trials and longitudinal cohort studies (29). In this sense, it would be convenient to explain to the families of children who are pre-myopic that they spend more time outdoors as a protection factor. However, this did not reduce myopia progression in children who already have myopia.

Regarding near-vision activities and time spent using electronic devices, in the pre-COVID period, myopes were the ones who spent more time compared to the other two groups. During the COVID period, no differences were found between all groups to near activities, but the percentage of time spent using electronic devices was higher in the pre-myopic children. These results obtained in our study with Spanish children differ from the results obtained in a group of American children with an average age of 8.3 +/−2.4 years, where based on parental reports, outdoor time decreased in myopic and non-myopic children, being the myopic children those who had a significantly lower level of daily light exposure and in relation with the electronic device the same study showed that both myopic and non-myopic children increased their use significantly during COVID-19 (19). The difference in results may be due to the difference in the average age of the studied children; our study included younger children (myopic: 6.25+/−0.77, pre-myopic: 6.11+/−0.79, hyperopic: 6.13+/−0.79). Children between 6 and 7 years of age are less likely to have unsupervised access to screen-based technologies (30) and spend more time outdoors and less time on near-work activities. Another possible reason for the differences in results could be that the questionnaires were administered at different periods after the COVID-19 pandemic in both studies. The questionnaires for our study after the COVID lockdown were carried out during the months of September and October 2020 and 2021, during the pandemic but after the lockdown when there were no longer mobility restrictions. In contrast, Mirhajianmoghadam et al. study (18) was conducted during the summer of 2020 while COVID-19-related quarantine measures were placed in Houston, the place where the study was done. The timing of data collection about the pandemic may have affected the results, as the restrictions and guidelines for outdoor activities and electronic device use may have changed over time. Therefore, it is important to consider the timing of data collection when comparing results from different studies. Although our results show that Spanish children after lockdown changed their habits by increasing time outdoors and decreasing the time for near vision activities and the use of electronic devices, some studies have reported an increased screen time and a decreased outdoor time among children during periods of strict COVID-19 regulations in other regions of the world (30–32). A recent systematic review (14) of the effects of remote learning during the COVID-19 lockdown on children’s visual health showed that most of the studies revealed a decline in visual health among children who were exposed to virtual learning strategies during the COVID-19 lockdown. Most of the studies specifically addressed the development and progression of myopia, indicating a faster onset and progression during the lockdown period related to the use of electronic devices (31), however, the systematic review by Lanca et al. suggests that the evidence is inconclusive and not convincing (32). These disparities underscore the significance of utilizing objective metrics to measure the time children spend on near-vision activities including electronic devices and the real time spent outdoors.

Our results show that the value of refractive error, in the pre-COVID era was more positive than in the COVID era. In both periods, the spherical equivalent was more positive with increasing outdoor time, and the spherical equivalent becomes more negative in both periods with increasing hours of near work and a higher percentage of electronic device use. These results are in line with two studies conducted with Chinese children in which was found a correlation between increased digital screen time and a greater change in SER during the COVID-19 pandemic (8, 33). If we analyze the percentage of use of electronic devices based on refractive error the myopic children in the pre-COVID period and the pre-myope children in the COVID period were the ones with the highest percentage of use. Digital devices have become a routine part of daily life in children in the last few years, using them both at school and home. A systematic review and meta-analysis suggest that excessive smart device use could be associated with myopia (32). These devices have been integrated into the education systems of numerous countries, in this sense The American Academy of Pediatrics recommended in 2016 that children between the ages of 2 and 5 should have no more than 1 h of screen time per day, and children over 6 years old should decrease their use of electronic devices (33). Even some countries have introduced laws to control the amount of time dedicated to digital screens in young children (32).

It is difficult to attribute the increase in myopia onset solely to the pandemic and remote learning, but it is known that the measures implemented to control the COVID-19 pandemic, such as lockdowns and social distancing, have accelerated the universal adoption of screen-based engagement globally. With limited access to outdoor activities and social interaction, people of all ages have increasingly turned to electronic devices for communication, entertainment, and education. Although the COVID-19 pandemic lockdowns had a generally negative impact on the development of myopia, due to a significant reduction in outdoor activities and an increase in near-work activities, our study shows that Spanish children between the ages of 5 and 7, once mobility restrictions were removed, increased their time outdoors and decreased their time for near vision activities and the use of electronic devices.

Myopia is one of the leading causes of visual impairment and blindness in many countries around the world. Preventing the onset of myopia is crucial in decreasing the prevalence of myopia in society. Early detection and intervention are important in managing myopia, and regular eye exams are recommended for children at risk of developing myopia. Some interventions that may be effective in the pre-myopia stage can include increasing outdoor time and reducing screen time. These interventions may help slow the progression of myopia and reduce the risk of developing high myopia, which can lead to more severe eye problems later in life.

In summary, there is enough evidence to support that spending more time outdoors is an effective method for preventing the development of myopia and slowing the myopic shift in refractive error in pre-myopic children and there is evidence that near activities and excessive use of digital devices could be strongly associated with myopia. In this sense, parents and caregivers must take measures to protect children’s eyes including limiting the use of electronic devices and promoting outdoor activity and exposure to sunlight. Interventions should be implemented as early as possible to slow the progression of the condition using pharmaceutical treatments or specially designed contact lenses or glasses that can help slow the progression of myopia.

Our study had some limitations; outdoor and screen time was based on self-reporting and not measured objectively. Further studies must be done with objective measurements of ambient light exposure and time spent with digital devices to eliminate bias. The methodology followed in the different years was the same. However, the refraction was non-cycloplegic, and SE might be overestimated. It should be noted that the data collection has been carried out using convenience sampling, which is why it presents an inability to generalize the results to the population, less representativeness of a specific population, and a greater probability of bias in the results. In turn, it has not been considered whether there are differences between workdays and weekends. Finally, due to the absence of a biometer, we cannot take the axial length as an objective variable to compare. This will have to be considered in future studies.

Due to the different COVID restrictions around the world, it would be needed for further studies to clearly explain how long after the lockdowns and restrictions have been the measurements taken. It is very difficult to make comparisons with other studies around the world if this data is unclear.

## 5 Conclusion

In conclusion, this research confirms that in the pre-COVID period, pre-myopic children were the ones who spent the most time outdoors, but after COVID all groups of children analyzed increased their time outdoors and pre-myopic children increased the use of electronic devices.

Further studies are needed to know if these lifestyle changes remain and how they influence the onset of myopia.

## Data availability statement

The raw data supporting the conclusions of this article will be made available by the authors, without undue reservation.

## Ethics statement

The studies involving humans were approved by April 25th, 2019, ethics committee of the European University of Madrid under code CIPI/19/102. The studies were conducted in accordance with the local legislation and institutional requirements. Written informed consent for participation in this study was provided by the participants’ legal guardians/next of kin.

## Author contributions

CA-P: Conceptualization, Data curation, Formal analysis, Funding acquisition, Investigation, Methodology, Project administration, Resources, Software, Supervision, Validation, Visualization, Writing – original draft, Writing – review & editing. AR-P: Visualization, Writing – original draft, Writing – review & editing. CM-P: Conceptualization, Data curation, Formal analysis, Funding acquisition, Investigation, Methodology, Resources, Software, Supervision, Validation, Visualization, Writing – original draft, Writing – review & editing. FP-G: Visualization, Writing – original draft, Writing – review & editing. CV-C: Conceptualization, Data curation, Formal analysis, Funding acquisition, Investigation, Methodology, Project administration, Resources, Software, Supervision, Validation, Visualization, Writing – original draft, Writing – review & editing. MG-P: Visualization, Writing – review & editing, Data curation, Funding acquisition. AG-A: Visualization, Writing – review & editing. MS-T: Data curation, Funding acquisition, Visualization, Writing – review & editing, Conceptualization, Formal analysis, Investigation, Methodology, Project administration, Resources, Software, Supervision, Validation, Writing – original draft.
